# Use of electronic dietary assessment tools in primary care: an interdisciplinary perspective

**DOI:** 10.1186/s12911-015-0138-6

**Published:** 2015-02-25

**Authors:** Carolina Bonilla, Paula Brauer, Dawna Royall, Heather Keller, Rhona M Hanning, Alba DiCenso

**Affiliations:** Department of Family Relations and Applied Nutrition, University of Guelph, Guelph, Canada; Nutrition Research Consulting, Fergus, Canada; Department of Kinesiology, University of Waterloo, Waterloo, Canada; School of Public Health and Health Systems, University of Waterloo, Waterloo, Canada; School of Nursing and Department of Clinical Epidemiology & Biostatistics, McMaster University, Hamilton, Canada

**Keywords:** Diet, Nutrition therapy, Nutrition assessment, Patient care team, Software, Websites, Mobile applications, Mixed methods

## Abstract

**Background:**

Dietary assessment can be challenging for many reasons, including the wide variety of foods, eating patterns and nutrients to be considered. In team-based primary care practice, various disciplines may be involved in assessing diet. Electronic-based dietary assessment (e-DA) instruments available now through mobile apps or websites can potentially facilitate dietary assessment. Providers views of facilitators and barriers related to e-DA instruments and their recommendations for improvement can inform the further development of these tools. The objective of this study was to explore provider perspectives on e-DA tools in mobile apps and websites.

**Methods:**

The exploratory sequential mixed methods design included interdisciplinary focus groups followed by a web-based survey sent to Family Health Teams throughout Ontario, Canada. Descriptive and bivariate analyses were completed. Focus group transcripts contributed to web-survey content, while interpretive themes added depth and context.

**Results:**

11 focus groups with 50 providers revealed varying perspectives on the use of e-DA for: 1) improving patients’ eating habits; 2) improving the quality of dietary assessment; and, 3) integrating e-DA into the care process. In the web-survey 191 respondents from nine disciplines in 73 FHTs completed the survey. Dietitians reported greater use of e-DA than other providers (63% vs.19%; *p* = .000) respectively. There was strong interest among disciplines in the use of e-DA tools for the management of obesity, diabetes and heart disease, especially for patient self-monitoring. Barriers identified were: patients’ lack of comfort with using technology, misinterpretation of e-DA results by patients, time and education for providers to interpret results, and time for providers to offer counselling.

**Conclusions:**

e-DA tools in mobile apps and websites may improve dietary counselling over time. Addressing the identified facilitators and barriers can potentially promote the uptake of e-DA into clinical practice.

## Background

Technology-assisted dietary assessment can improve the evaluation of food and nutrient intake in clinical practice. Six types of technology-assisted instruments for dietary assessment have been developed: interactive computer-based technologies, Personal Digital Assistants (PDAs), web-based technologies, mobile devices, specialized cameras and tape recorders, and scan and sensor technologies [1,2]. Many of these instruments have largely evolved from traditional paper-based methods that had people record the types and quantities of food eaten (e.g., food records, food frequency questionnaires, diet histories). Typically, these instruments have been validated through one-on-one client interviews, three- or seven-day food records, or video-recording food consumption [3-5].

The main advantages of these instruments are increased accuracy of food and nutrient intake information through inclusion of food photographs to assist in portion size estimation, direct data entry that reduces errors and interviewer bias, and immediate data analysis. Limitations include the need for a computer or mobile device, Internet connectivity, and familiarity with the software [6-8]. For the purpose of this study the term electronic dietary assessment (e-DA) will be used to refer to the use of web-based tools and mobile devices for dietary assessment [9-11].

Dietary counselling is an important activity in the prevention and treatment of multiple medical conditions and is often a key focus at certain life stages. For example, dietary counselling is common in the prevention and treatment of cardiovascular diseases, gastro-intestinal conditions, allergies, diabetes, unexplained weight loss, and cancer. In addition, pregnancy, infancy and the elderly are life stages when more focus on dietary intake may be warranted. Such counselling often occurs in primary care (PC), which refers to the first-contact of care with a health provider. Primary care deals with the majority of health problems in the population, and is the foundation of any healthcare system [12].

Team-based care is the preferred model of PC delivery in Canada. In Ontario, Family Health Teams (FHTs) have emerged in the past 10 years, and include physicians, nurse practitioners and nurses as well as other providers such as dietitians, pharmacists, and social workers according to the needs of the community [13]. In FHTs, dietitians support other team members who generally provide brief diet advice (e.g. non-prescriptive dietary guidelines) to clients during clinical visits. Clients identified as needing specialized dietary counselling are referred to team dietitians who provide in-depth individual and group behavioural counselling (e.g. using goal-setting and self-monitoring) to develop skills and motivation to undertake the specific diet changes [14,15]. Dietary counselling has clear goals with prescribed nutritive content and usually occurs over several encounters. Dietitians provide feedback to team members through medical record charting and/or meetings to reinforce dietary counselling goals [14]. FHTs use multiple electronic tools to support care and self-management activities by patients; however there is very little information on providers’ perspectives on the potential use of e-DA tools practice.

The aims of this study were to explore providers’ views on the potential use of e-DA tools within mobile apps and websites in an interdisciplinary PC context, to identify facilitators and barriers to greater use, and to provide recommendations for further development.

## Methods

The Ottawa Model of Research Use (OMRU), and specifically the subsection “assessment of potential adopters” [16], was chosen to guide the work. The exploratory sequential mixed methods design included first, interdisciplinary focus groups to describe current practices and conceptualize facilitators and barriers to e-DA tool use. Second, a web-based survey was conducted to assess practice among all available FHTs [17]. Focus groups were chosen over other methods of data collection to enable participants to describe individually and as a group their own experiences and perceptions on the potential use of the new technology [18].

### Focus group recruitment and sample size

A convenience sample of various provider disciplines participated in the multidisciplinary focus groups. Family physicians (FPs), registered nurses (RNs), nurse practitioners (NPs), registered dietitians (RDs) and clinical pharmacists (Phars) were especially targeted since they often provide nutrition counselling [14,15]. Previous experience using e-DA tools was not a criterion for participation because investigators wanted to obtain a range of perspectives. Executive directors of 102 FHTs and a local FHT-RD network were asked to distribute email invitations to their clinical staff. Recruitment was completed in three months. Participation was voluntary and participants were not remunerated for their time. Focus groups were offered during the lunch break to facilitate participation. To obtain sufficient interdisciplinary perspectives on the topic, it was anticipated that eight focus groups would be required to achieve theoretical saturation [19,20].

### Focus group interview guide

The focus group interview guide was developed in a multi-phase process. Initially, one-on-one interviews were conducted with 11 providers from diverse disciplines in two FHTs. During interviews it was noted that providers, other than RDs, were frequently not aware of e-DA tools. Thus, a short demonstration of screen shots of e-DA tools was considered important to ensure that all discussants could provide informed opinions on this topic. Results of these interviews were summarized and a semi-structured interview guide was created. The moderator presented the following script: “Electronic dietary assessment tools or e-DA tools use information technology to collect and analyse dietary information. Various e-DA tools exist, among them web-based dietary assessment tools and mobile applications (apps) in Smartphones and Tablets. I will pass along coloured paper copies of screen shots as examples of these tools but many more exist in the market. Please take your time to review these pages. Do you have any questions? After looking at the examples:

1. Were you aware of the existence of these tools? Which ones?
2. What would be the potential challenges of using e-DA tools in your clinic? What would be the potential benefits?
3. Could you provide us with recommendations or ideas on this topic?”

A pilot focus group was conducted involving four providers (FP, NP, RD and Phar); only one question required modification and this focus group was included in the analyses.

**Figure 1:**
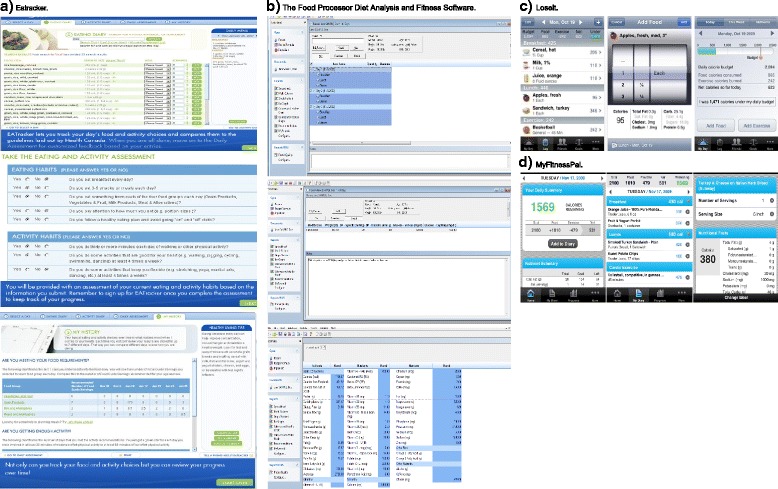
**Screen shots of electronic dietary assessment tools shown to research participants in focus groups. a)** Eatracker. **b)** The Food Processor Diet Analysis and Fitness Software. **c)** Loseit. **d)** MyFitnessPal.

Screen shots of four e-DA tools, as taken from their websites, were shown to participants at the beginning of each focus group. These included: the web-based tools Eatracker from Dietitians of Canada [21], The Food Processor Diet Analysis and Fitness Software [22], and the mobile apps Loseit [23] and MyFitnessPal [24] (Figure 1).

A trained moderator led all focus groups, which took place at each PC practice setting. A note-taker was present and field notes were summarized at the end of each session. Focus groups were digitally recorded and transcribed verbatim. To protect identity, names of participants and locations were removed; only the health provider role was retained.

### Focus group data analysis

The primary researcher (CB) attended all focus groups and reviewed each transcript for accuracy. Transcripts were analyzed using thematic analysis [25]. A three-person research team individually read transcripts in an “active way” searching for initial ideas and potential patterns. Some data were treated descriptively due to the nature of questioning, while more in-depth discussion involved greater interpretive analysis into themes. Descriptive data were summarized into tables and interpretive data were analyzed inductively and converted into codes; this formed the basis of emerging themes. A codebook was developed and the research team completed the coding; discrepancies were discussed and consensus was achieved. Two coders reviewed two randomly selected focus groups to complete the reliability of the codebook (18% of the sample). The two coders obtained a 90% agreement on their coding. Data saturation was observed from focus group six and on. Three focus group participants reviewed the results and provided feedback (member checking) to support credibility of results.

Trustworthiness is an important goal of qualitative studies of health services [26]; thus the perspectives of several disciplines across multiple sites within one PC model were sought to identify themes in common. Dependability was addressed in this study by using similar methods of data collection in each focus group and by use of multiple analysts and a consensus based approach to naming codes and themes.

### Web-based survey sample size and recruitment

Similar to the focus groups, invitations to participate were sent via executive directors of 112 FHTs. The invitation included a link to the SurveyMonkey® web-based survey posted online [27]. The survey included 18 closed- and open-ended questions that could be completed in about 10 minutes. Individual practitioners voluntarily completed the survey with no remuneration for their time. There was no minimum or maximum number of participants per team and it was available for a period of three months. Reminders were sent by email two times, one week apart.

As no previous similar surveys were found, a sample size calculation was done based on prevalence of dietary assessment assuming a two-sided, 95% confidence interval (CI) of ± 0.10 for a single proportion at 0.5. The sample size needed to asses prevalence of diet assessment was 97 overall, but to compare two proportions with a 0.05 two-sided significance level and 80% power would require more participants (nQuery Advisor 4.0). For example, 242 providers overall would be needed to compare subgroups where 50% of one group did diet assessment vs. 30% of another group.

### Web-based survey development

The analysis of focus groups provided content for development of the survey. After the thematic analysis, a draft questionnaire was created and the research team reviewed it for face and content validity taking into consideration the OMRU framework [28]. Thereafter, an online version was created and cognitive interviews with verbal probing were completed with six providers to assess the usability of the interface and understanding of the questions.

### Web-based survey analysis

Descriptive and bivariate analyses were completed. The data were first analyzed as a whole including all provider disciplines. Exploratory analyses of RDs versus other providers with less specific training in nutrition were also undertaken. Pearson’s chi-square test (*χ*^*2*^) followed by comparisons of proportions was used to examine relationships of the categorical data from the web-survey using SPSS software (IBM Corp, Version 20.0. Armonk, NY, 2011).

Research clearance was provided by the Research Ethics Board at the University of Guelph, Ontario, Canada. Informed consent was obtained from focus group and web-based survey participants before their involvement in the study.

## Results

In total, 50 providers participated in 11 focus groups. Each focus group was conducted at one FHT and included three to eight participants from different disciplines. The average duration was 56 minutes. Nine (82%) of participating FHTs were located in urban areas and two (18%) were in rural areas of the province. Most participants were female (80%) and (28%) were RDs. In comparison with the RD group, more of the other providers were in the older age group (≥36 years) and had 10 years or more of work experience (*p* = .000) (Table 1).

**Table 1:** **Demographic characteristics of participants**

	**Focus groups N = 50(%)**		**Web-based survey N = 191(%)**		
	**Registered dietitians**	**Other providers**	**Registered dietitians**	**Other providers**	***χ*** ^***2***^
**Number**	14 (28)	36 (72)	73 (38)	118 (62)	
**Age in years**					
<36	7 (50)	7 (19)	**45 (62)**	**28 (24)**	0.000
≥36	7 (50)	29 (81)	28 (38)	90 (76)	
**Gender**					
Male	1 (8)	7 (19)	1 (1)	21 (18)	
Female	11 (92)	29 (81)	72 (99)	97 (82)	
**Years in practice**					
0–< 10	8 (57)	9 (25)	**49 (67)**	**33 (28)**	0.000
10–25	6 (43)	27 (75)	24 (33)	85 (72)	

Statistically significant differences between two variables are in bold numbers. Percentage (%) is within health provider group.

For the web-based survey, 73 (65%) of the 112 FHTs known to have received an invitation had at least one respondent. Of these, 55 (75%) FHTs were located in urban areas and 18 (25%) in rural areas of the province. Although 231 individuals completed the survey, 191 respondents were direct care providers, of which 73 (38%) were RDs and 118 (62%) were other providers. The remaining questionnaires (n = 40) were completed by non-clinical care staff and were excluded from the analyses. Participants were predominantly female (88%), and in comparison to other providers, more RDs were in the younger age group (<36 years) and had less than 10 years of work experience (Table 1). Most FHTs had a RD (83%), but 12 (17%) did not have a RD on staff.

A range of experience with e-DA tools was observed in the interdisciplinary focus groups. Insights emerged under three main themes: improving patients’ eating habits, improving the quality of dietary assessment, and integration of e-DA tools into the care process. Quotes from providers are identified by discipline and focus group, in addition to whether providers were currently using e-DA tools [e.g., e-DA tool user]. Sample quotes identified by citation number are shown in Table 2.

**Table 2:** **Focus group discussion themes and example quotes**

**Theme 1: Improving patients’ eating habits**		
**Subtheme**	**Citation**	**Quotes**
Raising awareness of diet	1	*“It is meant to be a tool for awareness. Most people come back and go - holy smokes! I had no idea I was doing this or doing that. It is an eye opener” (RD/FG#11) [e-DA tool user].*
	2	*“[it] Puts the responsibility back onto the patient, and identifies more what they’re eating and how their activities are working” (NP/FG#2).*
Increasing patients’ motivation	3	*“I think they would be motivated to see those patterns and colourful graphs” (RD/FG#1).*
	4	*“And they’ve a sense of self-control, because -‘Oh! Look what happens when I take out a teaspoon of sugar every day. I have lost some calories. Now, look at the carbs and my blood sugars improving-“(RD/FG#9) [e-DA tool user].*
	5	*“I think that it is also an assessment tool of how motivated people are, because if they are going to do it, then, that shows that they are ready to take that next step” (RD/FG#2) [e-DA tool user].*
**Theme 2: Improving the quality of dietary assessment**		
**Subtheme**	**Citation**	**Quotes**
Increasing the quantity and quality of dietary information	6	*“It’s a new tool and is very visual…I would explain [to] patients in a way that they could visualize why we are saying –‘You’re not getting enough fruits and vegetables’- or –‘You need more calcium in your diet’ “(RD3/FG#3).*
	7	*“Even in a normal visit, you might have somebody say: - ‘What is a recommended daily intake of…?’-I might not know off the top of my head” (Phar/FG#4) [e-DA tool user].*
Improving efficiency	8	*“If I could only convince them to do it for three days to get a really good summary and when they actually come in to see me we can look at it and have a thorough assessment” (RD/FG#1).*
**Theme 3: Integrating e-DA into the care process**		
**Subtheme**	**Citation**	**Quotes**
Balancing electronic media with face-to-face interactions	9	“I use a lot of email with my patients, and there is no reason why they shouldn’t just send me an email with their findings…you keep track and we will meet again…maybe easier for the patient” (RD1/FG#11) [e-DA tool user].
	10	“We haven’t opened up our office to email access by our patients; patients don’t often realize which is the most appropriate venue to be using our services” with patients to be attractive: (FP/FG#7).
	11	*“I think it could be a good element, but it’s not going to help unless there is some kind of counselling involved here” (NP/FG#2).*
Tailoring the e-DA tool to counselling	12	*“The dietary device needs to have the ability to print off easily and get the big picture and see patterns immediately instead of looking day by day” (RD1/FG#11) [e-DA tool user].*
	13	*“I don’t talk calories, I like the idea of you look at your meal and see what is balanced or not, especially when I work with young girls” (NP/FG#2) [e-DA user].*
	14	*“I am going to be communicating with people… the aspect of security or firewall protection I think is important” (RD/FG#7) [e-DA tool user].*

### Improving patients’ eating habits

Participants’ stated that e-DA tools could be used to create awareness of food intake and increase patient motivation for dietary self-monitoring, and consequently improve their eating habits.

### Raising awareness of diet

Participants noted that e-DA tools could be effective in helping patients become aware of the quantity and quality of foods consumed over time [citation 1]. Additionally, participants believed e-DA tools could be useful to support collaboration between the patient and provider in their care [citation 2]. By self-monitoring of diet, people can become more attentive and educated in their own disease condition and how diet influences outcomes. This empowers patients to make informed decisions on what, how much and how often to eat, and ultimately adopt healthier eating habits.

### Increasing patients’ motivation

The lack of motivation for dietary self-monitoring and dietary changes was identified as a major problem in conducting dietary assessment, monitoring, and treatment. RDs mentioned that, on average, one in five patients completed paper-based food records. Participants noted that e-DA tools could provide convenient access to diet information at any time and almost anywhere with a high degree of privacy, as long as the necessary technology is available. This may lead to an increase in patients’ dietary self-monitoring and sense of self-efficacy. Furthermore, patients may feel motivated to use e-DA tools more than traditional paper records due to the novelty and trendiness of mobile devices [citation 3]. In addition, the ability to make a logical connection between dietary modification and goal achievement was seen as a way of providing patients with a sense of self-control over their condition [citation 4].

Participants also commented that using e-DA tools could be an indicator of how motivated a patient is to adopt dietary recommendations [citation 5]. This participant further suggested that, in her experience, patients who complete dietary assessments are more receptive and want nutrition-related resources and services.

### Improving quality of dietary assessment

Participants noted that e-DA tools could also increase providers’ understanding of patients’ diets, which facilitates counselling. Improving the quality and quantity of dietary information obtained from patients, improves the quality of dietary assessment and promotes efficient counselling.

### Increasing the quality and quantity of dietary information

Participants noted that e-DA tools provided summaries of individual results presented as graphics and tables. They believed that this level of detail would facilitate providers’ discussions with patients during consult visits [citation 6]. In addition to these visual representations of diet quality, the ease with which data are entered and analyzed contributes to a large quantity of dietary data that can be used in assessment. Providers noted that with the support of e-DA results, they would be able to determine changes in eating patterns from weekdays to weekends, identify portion sizes, the frequency of skipped meals, eating out, and other eating patterns such as “the 10:00 o’clock trip to the refrigerator” (FP/FG#6). Since some e-DA tools also provide details of the recommended food guide servings, it was believed this comparison would be useful for non-RD providers [citation 7]. Thus, e-DA tools can facilitate patients’ visits because consults may be more interactive and data-driven.

### Improving efficiency

With the use of e-DA tools, providers can save time in completing and analyzing dietary information [citation 8]. With the instantaneous analysis achieved with e-DA, the provider can dedicate more time to nutrition education, and setting dietary strategies and goals. Additionally, clinical pharmacists emphasized that their patients often overuse supplements, and e-DA tools can be used to provide advice on whether to use or not use supplements in a more time-effective manner by obtaining a complete on-line food record that has been analyzed at the level of nutrients.

### Integration of e-DA into the care process

Focus group participants could envision the use of e-DA in their practices. Yet, to make the most of these tools, they need to be integrated into the care process with consideration given to how they can be balanced with face-to-face interactions and support, rather than replace counselling.

### Balancing electronic media with face-to-face interactions

Many providers liked the idea of accessing e-DA results electronically using the web based e-DA tool page, e-mail or their web portal as it allowed for a convenient and rapid way to communicate with patients [citation 9]. Moreover, some providers liked the idea of integrating these tools into the electronic medical record (EMR) system in a way similar to other current e-tools that track patients’ blood pressure or glucose levels. However, not all team members considered electronic communication with patients to be attractive, and avoided electronic communication with patients [citation 10]. As an alternative, some participants suggested that patients use e-DAs and bring their results to their clinic appointment, while others were concerned about having enough time to review patients’ information during clinical visits.

Integration of e-DA into the clinic consultation was discussed. Providers emphasized the importance of delivering personal guidance [citation 11]. Without sufficient guidance from a health professional there is a risk of “losing the patient within the e-DA tool” (RD2/FG#11) [e-DA tool user]. Specifically, if patients consistently receive negative feedback from e-DA results about not meeting their personal goals (e.g., lowering sodium in diet), it could lead to frustration, apathy, and noncompliance with treatment plans.

### Tailoring the e-DA tool to counselling

During the focus groups, participants viewed several e-DA platforms. They provided feedback on the overall e-DA tool format and identified the elements that could best suit their practice. The majority of participants noted that it was important to have an e-DA tool that identifies dietary patterns by matching the patient’s diet with well-known nutrition guidelines such as the DASH diet or a national food guide as this facilitates nutrition counselling with patients [citation 12] [29,30]. Most providers without a nutrition background preferred a basic dietary assessment focusing on information such as number of food groups, portion sizes, and preparation of food at home or eating out. In addition to this information, some RDs wanted a more detailed dietary assessment with specific details on macronutrients and micronutrients.

The population of patients seen in PC is varied. Thus, providers were interested in having an e-DA tool that could reach different audiences, such as adults and children. Of particular importance was that the e-DA tool be easily accessible, free of cost for patients, have web-based and mobile options, and be easy to complete requiring little or no assistance from a health provider. Participants noted that e-DA also provided an opportunity to gather information on other behaviours associated with poor diet such as physical inactivity, smoking, sleep habits and mood.

Being able to individualize results to specific clients was also desired. For example, one RD mentioned that focusing on calories could be problematic, especially in young people, women with eating disorders, or those with weight issues [citation 13]. As a result, it was suggested that having the option of omitting calories and other components as needed from the e-DA results would help to reinforce the key messages and approaches used in counselling by individual practitioners.

Lastly, if e-DA information is to be used in counselling individuals, it needs to be securely accessed with a protected password to ensure privacy of information and patient confidentiality: [citation 14]. Participants also stated that results obtained from e-DA tools must be compatible with the most common application software programs (e.g., Microsoft Office, Adobe Reader) that are typically used in their practices.

### Web-based survey results

The descriptive results from the focus groups were used to develop the web-based survey items. Of 181 respondents, 36% reported using e-DA tools to assess patients’ diets (Table 3). A higher proportion of RDs compared to other providers reported using e-DA tools with patients (*p =* .000). EaTracker, a web-based tool from Dietitians of Canada and Calorie Counter by MyFitnessPal, a web-based and mobile app, were the most frequently used e-DA tools. In comparison to other providers, a higher proportion of RDs reported patient use of e-DA tools (*p* = .000). As reported by participants, patients use a wide range of other e-DA tools, including: The Food Processor Diet Analysis and Fitness Software [22], My Plate Calorie Tracker [31], Weight Watchers [32], Sparkpeople [33], and a calcium calculator [34].

**Table 3:** **Web-based survey: provider reported use of e-DA tools**

	**All participants N = 181 (%)**	**Registered dietitians N = 73 (%)**	**Other providers N = 108 (%)**	***χ*** ^***2***^
I (provider) am/was using one of the following with patients (Check all that apply):				
I am using a web-based or mobile app for dietary assessment with my patients	65 (36)	**46 (63)**	**21 (19)**	0.000
eaTracker (Dietitians of Canada)*	34 (18)	32 (44)	2 (2)	
Calorie counter myfitnesspal**	20 (11)	**14 (19)**	**6 (5)**	0.006
Other (s) tools	7 (4)	6 (8)	1 (1)	
My patients are/were using one the following (Check all that apply):				
Patients are not using a web-based or mobile app for dietary assessment	76 (42)	**18 (25)**	**58 (54)**	0.000
calorie counter myfitnesspal**	73 (40)	**50 (68)**	**23 (21)**	0.000
eaTracker (Dietitians of Canada)*	55 (30)	**45 (62)**	**10 (9)**	0.000
Weight watchers**	37 (19)	19 (26)	18 (17)	
Calorie tracker (by Livestrong.com)**	20 (11)	12(16)	8 (7)	
Calorie count**	19 (10)	12 (16)	7 (6)	
LoseIt**	19 (10)	17 (23)	2 (2)	
Other tools	16 (9)	14 (19)	2 (2)	

Statistically significant differences between two variables are in bold numbers. Percentage (%) of total in columns is more than 100% due to multiple responses. Presentation of the tool: *web-based tool **web-based and mobile app tool.

The potential benefits of e-DA tools most reported by the respondents were: self-monitoring of nutrients (87%), an educational tool to allow self-reflection about diet (85%), and to motivate people to track their diet (84%). Very few providers saw no benefits of e-DA (3%) (Table 4). Notably, a higher proportion of RDs in comparison to other providers identified the following benefits of e-DA: potential for self-monitoring of nutrients, foods and eating behaviours, motivating people to track what they eat, and helping to track specific nutrients.

**Table 4:** **Web-based survey: facilitators and value of using e-DA tools in team-based care**

	**All participants N = 177 (%)**	**Registered dietitians N = 73 (%)**	**Other providers N = 104 (%)**	***χ*** ^***2***^
Potential benefits of using e-DA tools in my practice (Check all that apply):				
Potentially be used for self-monitoring of nutrients, foods and eating behaviours	154 (87)	**69 (95)**	**85 (82)**	0.013
An educational tool because they may allow patients to self-reflect about their own diet	150 (85)	65 (89)	85 (82)	
Motivate people to track what they eat because of rapid and visual results	148 (84)	**67 (92)**	**81 (77)**	0.014
Facilitate initial assessment of food intake and/or eating behaviours	117 (66)	46 (63)	71 (68)	
Help in tracking specific nutrients (e.g., vitamin K, calcium, sodium, potassium)	107 (63)	**54 (74)**	**53 (51)**	0.002
Provide more accurate results vs. paper records; e.g., food photographs, portion sizes, and assessment of food habits	100 (56)	**48 (66)**	**52 (50)**	0.030
Provide more detailed information on diet than is currently available	69 (39)	33 (45)	36 (35)	
Decrease time and cost of personnel in conducting dietary assessments	63 (36)	28 (38)	35 (34)	
No benefits	6 (3)	1 (1)	5 (5)	
New e-DA tools could be valuable in (Check all that apply):	N = 178 (%)	N = 73 (%)	N = 105 (%)	
Overweight/obesity without other conditions	168 (94)	71 (97)	97 (92)	
Diabetes with or without other conditions	165 (93)	68 (93)	97 (92)	
Heart disease	142 (80)	63 (86)	79 (75)	
General health promotion over the lifecycle (e.g., pregnancy, children, women)	138 (78)	53 (73)	85 (81)	
Gastrointestinal issues	123 (69)	52 (71)	71 (68)	
A combination of dyslipidemia, hypertension, not including diabetes or heart disease	111 (62)	47 (64)	64 (61)	
Wellness check-ups or annual physical examinations (adults or children)	104 (58)	**30 (41)**	**74 (71)**	0.000
Cancer	63 (35)	20 (27)	43 (41)	
Other condition (s)	13 (7)	8 (62)	5 (38)	

Statistically significant differences between two variables are in bold numbers. Percentage (%) of total in columns is more than 100% due to multiple responses.

Respondents identified e-DA tool use as valuable in overweight/obese patients (94%) and individuals with diabetes (93%). A higher proportion of other providers compared with RDs identified that e-DA tools could be useful for wellness check-ups (*p* = .000) (Table 4). Other conditions for which e-DA was seen to be useful were: allergies, smoking cessation, eating disorders, appetite changes due to depression or anxiety, arthritis, hepatitis C, celiac disease, vegan diets, osteoporosis, fatigue, headaches, and renal disease (data not shown). Survey participants reported the perceived barriers to e-DA tool use, including a lack of motivation for patients to complete dietary assessment (Table 5). A higher proportion of other providers compared with RDs identified that barriers to using e-DA tools were spending time and education to interpret results, and the time needed to offer counselling (both *p* < .01). In contrast, a higher proportion of RDs compared to other providers identified that barriers included: a lack of patient comfort with using of technology, low validity/reliability of e-DA tools (both *p *< .01), misinterpreting results by patients, and risk of foods not listed in the database (both *p *< .001). Other barriers expressed by participants were incorrect data entry (e.g., correct portions, specific foods/brands, combination meals), lack of ethnic/traditional foods and dishes in the database, clients not sufficiently fluent in speaking/written English, and an aging population uncomfortable with using technology (data not shown).

**Table 5:** **Web-based survey: barriers to using e-DA tools**

	**All participants N = 177 (%)**	**Registered dietitians N = 73 (%)**	**Other providers N = 104 (%)**	***χ*** ^***2***^
Potential barriers of e-DA tools use in my practice (Check all that apply):				
Lack of motivation by patients to complete dietary assessment	141 (80)	56 (77)	85 (82)	
Patients’ lack of comfort with use of technology	133 (75)	**56 (77)**	**57 (55)**	0.003
Time for patients to fill out the e-DA questionnaire	115 (65)	48 (66)	67 (64)	
Time in training patients to use e-DA tool to obtain more accurate results	109 (62)	46 (63)	63 (61)	
Cost of tool to your organization ($500–700 total/year)	91 (51)	33 (45)	58 (56)	
Inability to download dietary data directly into EMR	86 (49)	34 (47)	52 (50)	
Misinterpretation of results by patients (e.g., day-to-day variability of the diet)	83 (42)	**45 (62)**	**38 (37)**	0.001
Time and education for providers to interpret results at their offices	75 (42)	**22 (30)**	**53 (51)**	0.006
Validity/reliability of e-DA tools	75 (43)	**41 (56)**	**34 (33)**	0.002
Time for provider to offer counselling	73 (41)	**20 (27)**	**53 (51)**	0.002
Foods not listed in the database	60 (34)	**41 (56)**	**19 (18)**	0.000
Provider compensation of nutrition advice	40 (23)	**11 (15)**	**29 (28)**	0.045
Patients disclosure of diet information	37 (21)	14 (19)	23 (22)	
Misinterpretation of results by providers (e.g., the day-to-day variability of the diet)	34 (19)	11 (15)	23 (22)	
Safety and confidentiality issues	33 (19)	17 (23)	16 (15)	
No barriers	1 (1)	0 (0)	1 (1)	

Statistically significant differences between two variables are in bold numbers. Percentage (%) of total in columns is more than 100% due to multiple responses. EMR: Electronic Medical Record. Percentage (%) of total in columns is more than 100% due to multiple responses.

In the focus groups participants articulated that age of patients should not have an influence on e-DA tool use: “I think we are discriminating if we say: -‘Oh you’re old, so you’re not on the Internet’- 87-year old mothers are on the Internet, so we can’t do that ageism” (RD). Patient comfort with technology and easy-to-use tools was considered important factors for use.

Interest in uptake of e-DA in practice was high as 89% of participants stated that they would be open to learning more about e-DA tools. Recommendations to facilitate adoption of e-DA tools were provided and included space to indicate current disease (s) and medication (s) taken by patients; adding soluble and insoluble fiber and glycemic index values of foods; including options for use of voice and video camera to describe uncommon foods; and adding the top five recommendations based on dietary assessment for the patient’s nutrition-related condition.

## Discussion

While many developers have designed a wide range of nutrient analysis websites and apps for use by the public and researchers have been focused on clinical trials evaluating these resources, little is known about the potential for uptake of e-DA in the PC system and by healthcare teams. To our knowledge this is the first study addressing this gap.

The results reveal a substantial range of provider experience with e-DA, and as expected, the RD group reported both more experience in using e-DA tools and additional concerns about reliability, validity and inappropriate use in persons with eating disorders. Two major benefits of e-DA tools can be drawn from the study: providers believe that e-DA tools could help motivate and educate patients to adopt healthy eating habits, and e-DA tools would give providers a better understanding of patients’ diets and therefore the ability to offer better advice.

The vast majority of participants, including non-e-DA tools users, identified that these tools could assist patients in dietary self-monitoring, increase awareness of dietary intake and motivate them to adopt healthier diets. Behavioural self-monitoring, including diet and physical activity, is a key predictor of successful behavioural change [35]. The importance of self-monitoring is that it encourages people to pay attention to their own actions, the circumstances in which their actions occur, and the immediate and long-term consequences of these actions. According to the Social Cognitive Theory, perceived self-efficacy (the beliefs in one’s capabilities to execute the course of action required) and motivational support are bi-directional; with increases in either factor, the other factor is also improved [35,36]. Thus, e-DA tools might contribute to motivational support and therefore perceived self-efficacy in diet change.

According to previous research, when dietary assessment is supported with websites and mobile devices, the acceptability, user satisfaction, and adherence to dietary self-monitoring is superior compared to using paper records only. This is especially consistent in overweight and obese individuals participating in weight loss programs, and in people with type 2 diabetes or food allergies [4,6,37-41].

In overweight and obese individuals a combination of a nutrition intervention and dietary self-monitoring with e-DA tools has been shown to improve adherence to dietary tracking, which has then been associated with increased weight loss, and decreased waist circumference and body fat [6,11,40]. However, weight loss is challenging, and other researchers have not found differences in weight loss between paper-based and electronic dietary records [41]. Independent of the method, dietary self-monitoring adherence seems to decline over time. Consequently, close guidance by a health professional is recommended especially in people using electronic tools to promote dietary change [6]. With the assistance of a health professional, e-DA could facilitate dietary self-monitoring, enhance the understanding of nutritional values of certain foods and eating patterns, and support the establishment of personal goals, and eventually dietary change.

The new generation of apps in mobile devices are highly popular worldwide with multimedia capabilities including telephone, Short Message Service (SMS), photos, music, video, and social media [42]. Health apps for mobile devices provide convenient access to information any time and almost anywhere in a private environment. Hence, it is expected that these devices will continue to be popular and become an accepted method to gather medical information as well as deliver care [43]. Factors such as acceptability and comfort with technology were identified in our study as being more important than the patient’s age, which is consistent with other reports [11,44].

For providers, a strong facilitator for using e-DA in practice was the improvement in dietary information quality and efficiency. For instance, real time food intake data are automatically calculated and nutrient content is reported in colourful presentations. Some e-DA tool results deliver food serving recommendations according to a national food guide, which is useful in discussing eating patterns [21,45]. The majority of e-DA tools contain large food databases that include foods from restaurants and fast food chains. The reporting of food consumed is easier and more accurate since most tools incorporate immediate checks for incomplete responses and use pictures for portion sizes. As there is no need for a health practitioner to complete the assessment, it is believed that users may feel more comfortable to report actual food consumption [45-47]. Moreover, closer contact with the health provider via a mobile device may increase the assurance and accountability of patients, and providers may deliver better clinical decisions with more precise and timely information [48].

To date, limited studies have been published to confirm these assertions with the range of patients seen in PC. Two studies in PC, one of general consultations and one of patients with type 2 diabetes [9,10], reported that dietitians spent less time in nutrition consultations and more time on diet education and counselling when clients used a web-based dietary assessment tool. Additional studies are needed to confirm these promising results.

Multiple barriers need to be addressed before implementation of e-DA tools in team-based PC. Many of the potential barriers identified in both focus groups and web-based surveys are consistent with the literature [6,44,48-50]. An additional barrier for patients identified in our study was the inappropriate use of e-DA tools, which could perpetuate extreme or obsessive calorie counting in some individuals with eating disorders. The prevalence and severity of such problems are currently unknown and warrants further work. In certain individuals, having an option to hide calorie information and monitoring could help to address this issue.

The web-survey investigated the use of e-DA tools in clinical practice. RDs reported higher use of e-DA tools than other providers, but use is still low. In a recent publication, 40.5% of RDs reported recommending nutrition/food apps to clients in their practice, most often for weight loss/obesity, healthy eating and diabetes [50]. However, the lack of adaptability of the current generation of healthcare apps, including e-DA tools, for use in clinical environments can partially explain low usability [43,50]. Additional development, taking into account team members’ dietary counselling needs, practice needs, preferences and routines should lead to improved functionality over time.

The difficulty that patients experience in disclosing dietary information to providers can be a limitation in dietary assessment [3]. One important reason for this is patient-perceived stigmatization. In a systematic review of interventions delivered through the Internet, it was found that electronic systems offer privacy and a reduction of stigmatization, especially in those with chronic conditions such as mental illness, diabetes, and women with eating disorders [51]. These conditions are nutrition-related and are often seen in PC, thus using e-DA tools in these individuals can offer a new alternative [52]. Another barrier is the medico-legal implications of using mobile devices in PC. For example, if a provider does not respond to a patient immediately and the patient’s health deteriorates. The medico-legal implications of using mobile devices warrant further investigation [48].

Other barriers identified in this study were e-DA tool incompatibility with EMRs, potential misinterpretation of results by providers, and unknown validity and reliability of many of the e-DA apps. It has been reported, however, that training and continuous support of providers are key elements needed to increase providers’ knowledge, confidence and acceptance of technological devices in clinical settings [53,54].

Clinicians providing nutrition services in team-based care made multiple recommendations. For successful implementation of an innovation in PC, the new tool must be easily accessible, suit providers’ day-to-day practice, and provide a certain level of comfort [55,56]. For example, when web-based tools were utilized for the management of diabetes and cardiovascular disease, the main factors that increased adoption in clinical practice were the usefulness, usability and sustainability of the instrument [57]. Similarly, participants in our focus groups stated that they would recommend an e-DA tool only if it was user-friendly, easily accessible, free of cost, and guided patients to complete dietary assessments electronically with little or no assistance from a health provider. Participants also wanted a variety of interactive options customized to the characteristics of the team-based organization, provider preferences and patient needs. However, a group of participants preferred that only patients use e-DAs and bring their results to a consultation. To increase the usability in less technology savvy populations, the use of “tutorials” and “help” buttons, text enlargement options, spelling aids, free-text option boxes, and navigation bars have been recommended [49].

Participants in this study identified Calorie Counter by MyfitnessPal app as the most frequently used e-DA tool for patients. In a recent publication, this diet app was also identified as the most frequently used calorie counter and fitness tracker app in the United States [43]. Although this tool was not designed for PC, it is free of cost, has web-based and mobile device options, an extensive food database, and offers “peer support” by adding users’ friends to participate in blogs and chat rooms. While accuracy has been questioned, the popularity of its features warrants consideration. Two literature reviews in PC settings found that online peer support involving friends and their providers was key to successful Internet-based health promotion interventions [58,59]. Likewise, when using mobile apps in patients with diabetes, the information should be updated, interactive, offer feedback, and provide the option of peer support [60].

Participants noted a high level of interest in using e-DA tools particularly in the management of excess weight and obesity, cardiovascular disease, diabetes, mood disorders, allergies and inflammatory conditions including arthritis, and wellness check-ups. This finding echoes what others have reported [50]; these diseases are prevalent in PC settings [52] and can be prevented or managed with lifestyle changes, including diet [15].

In interdisciplinary practice, some patients consider the use of technological devices as an opportunity to be informed and supported outside of a health organization [55,59] and may seek providers’ advice on electronic tool recommendations. Therefore providers must become familiar with these types of tools. Additional studies are needed examining the use of e-DA and their impact on overall medical care [43].

### Strengths and limitations of the study

The use of a strong theoretical framework, specifically the OMRU, the sequential mixed methods study design, and focus on team perspectives were all strengths of our study. In contrast to many studies that create homogeneous focus groups by socio-demographic characteristics, we deliberately brought different professions together to discuss a shared issue and confirm and extend the knowledge of facilitators and barriers to e-DA use in PC. Rigour of data collection and analysis enhances the trustworthiness of these findings [26]. The interest of diverse professions who participated in our study highlights the importance of nutrition in PC practice [15,53] and likely of greatest importance, their personal interest and stake in this topic. Thus, results from this study incorporate diverse perspectives and useful information for future developers.

There are several limitations of the study. First, perspectives of participants were assessed, not actual behaviour of providers. It is also likely that the sample was not representative of all FHT providers, and consisted of interested counsellors or early adopters. As this is one of the first studies of provider perspectives, it is reasonable to gather views of the interested providers first. The actual response rate could not be calculated, as information on the number of providers in all contacted FHTs was not available. The e-DA tools listed in the questionnaire could be limited to the region and time of the study. Furthermore, this study collected information from providers only, and the opinions of patients and administrative staff as potential users of e-DA tools remain unknown.

## Conclusions

According to the results of this study, facilitators of e-DA tool use for patients include support in dietary self-monitoring, increased awareness of dietary intake, and motivation to adopt healthy eating habits. For providers, facilitators included improvement in patient dietary information and efficiency in assessing diet. There was strong interest among all disciplines in the use of e-DA tools for the management of obesity, diabetes and heart disease. The most important barriers for e-DA tool use in the RD group were the lack of patient comfort with use of technology, low validity/reliability of e-DA tools, potential misinterpretation of results by patients, and risk of foods not available in the database. In contrast, identified barriers for other providers were spending time and education to interpret results and offer counselling, and provider’s compensation for nutrition advice.

Due to the interdisciplinary context of the study, our results provide insight into opportunities for increasing knowledge of e-DA tools by providers and adapting these tools to suit practice settings. The adaptability and usability of e-DA tools in clinical environments should be considered in consultation with providers, according to their particular needs, preferences, and available resources. The specific recommendations made by participants provide valuable information for incorporating specific features in the next generation of e-DA tools.

## Abbreviations

CCM: Chronic care model
e-DA: Electronic-based Dietary Assessment
FHT: Family Health Team (s)
NP: Nurse Practitioner (s)
Phars: Pharmacist (s)
OMRU: The Ottawa Model of Research Use
PC: Primary Care
Phar: Pharmacist (s)
FP: Family Physician (s)
RD: Registered Dietitian (s)
RN: Registered Nurse (s)
EMR: electronic medical record
app: application
